# The Host Cell Metabolite Inositol Hexakisphosphate Promotes Efficient Endogenous HIV-1 Reverse Transcription by Stabilizing the Viral Capsid

**DOI:** 10.1128/mBio.02820-20

**Published:** 2020-12-01

**Authors:** Jordan Jennings, Jiong Shi, Janani Varadarajan, Parker J. Jamieson, Christopher Aiken

**Affiliations:** a Department of Pathology, Microbiology and Immunology, Vanderbilt University Medical Center, Nashville, Tennessee, USA; University of Pittsburgh School of Medicine

**Keywords:** HIV-1, IP6, capsid, reverse transcription, uncoating

## Abstract

A defining activity of retroviruses is reverse transcription, the process by which the viral genomic RNA is converted into the double-stranded DNA required for virus replication. Reverse transcriptase (RT), the viral enzyme responsible for this process, was identified in 1970 by assaying permeabilized retrovirus particles for DNA synthesis *in vitro*. Such reactions are inefficient, with only a small fraction of viral genomes being converted to full-length double-stranded DNA molecules, possibly owing to disruption of the structure of the viral core. Here, we show that reverse transcription in purified HIV-1 cores is enhanced by the addition of the capsid-binding host cell metabolite inositol hexakisphosphate (IP6). IP6 potently enhanced full-length minus-strand synthesis, as did hexacarboxybenzene (HCB), which also stabilizes the HIV-1 capsid. Both IP6 and HCB stabilized the association of the viral CA and RT proteins with HIV-1 cores. In contrast to the wild type, cores isolated from mutant HIV-1 particles containing intrinsically hyperstable capsids exhibited relatively efficient reverse transcription in the absence of IP6, further indicating that the compound promotes reverse transcription by stabilizing the viral capsid. We also observed that the capsid-destabilizing antiviral compound PF74 inhibited endogenous reverse transcription with a potency that mirrors its ability to inhibit reverse transcription during infection. Our results show that the stabilization of the HIV-1 capsid permits efficient reverse transcription in HIV-1 cores, providing a sensitive experimental system for analyzing the functions of viral and host cell molecules and the role of capsid disassembly (uncoating) in the process.

## Abstract (precis)

HIV-1 infection requires reverse transcription of the viral genome. While much is known about the biochemistry of reverse transcription from simplified biochemical reactions, reverse transcription during infection takes place within a viral core. However, endogenous reverse transcription reactions using permeabilized HIV-1 virions or purified viral cores have been inefficient. Using viral cores purified from infectious HIV-1 particles, we show that efficient reverse transcription is achieved *in vitro* by addition of the capsid-stabilizing metabolite inositol hexakisphosphate. The enhancement of reverse transcription was linked to the capsid-stabilizing effect of the compound, consistent with the known requirement for an intact or semi-intact viral capsid for HIV-1 infection. Our results establish a biologically relevant system for dissecting the function of the viral capsid and its disassembly during reverse transcription. The system should also prove useful for mechanistic studies of capsid-targeting antiviral drugs.

## INTRODUCTION

During retrovirus infection, the viral membrane fuses with the target cell, releasing the viral core into the cytoplasm. The core, consisting of a capsid shell surrounding the viral genome and its associated proteins, is the functional viral payload. In the cell, the viral RNA genome is converted into a double-stranded DNA molecule by reverse transcription, producing the *cis*-acting viral sequences necessary for integration and subsequent gene expression. Reverse transcription is catalyzed by the viral reverse transcriptase enzyme (RT) and takes place in a ribonucleoprotein complex housed within the viral capsid. For HIV-1, pharmacological or genetic perturbations of the stability of the capsid typically result in impaired infectivity. Specifically, destabilization of the viral capsid leads to inefficient reverse transcription in target cells (1, 2), while hyperstabilization of the capsid inhibits nuclear entry and integration (3, 4). Similarly, premature capsid disruption in cells expressing restrictive TRIM5 proteins is associated with impaired reverse transcription (5, 6). Collectively, these studies have established that the integrity of the viral capsid is important for efficient HIV-1 reverse transcription in target cells.

HIV-1 reverse transcription occurs in a series of stages (reviewed in reference 7). Synthesis of the minus strand is primed near the 5′ end of the genome by a tRNA, resulting in runoff synthesis of a short DNA molecule, the minus-strand strong stop. Subsequently, this product anneals to the 3′ end of the genome and is extended, resulting in an ∼9-kb minus-strand product. Plus-strand synthesis is primed by a small RNA remnant at the beginning of the U3 sequence, resulting in a short product that is then extended after annealing to the 3′ end of the minus strand. Subsequently, synthesis of the two viral long terminal repeat sequences (LTRs) is completed, resulting in a preintegration complex (PIC) that catalyzes the integration of the nascent viral DNA into the target cell genome. A recent study suggests that nuclear entry precedes the completion of reverse transcription, suggesting that the core/reverse transcription complex responds to a specific nuclear signal or is activated during the process of nuclear entry (8). Nonetheless, it is known that active PICs containing two complete DNA ends can be recovered from the cytoplasm of acutely infected cells (9).

Inositol phosphates are abundant cellular metabolites that participate in a wide array of cell activities (reviewed in reference 10). These highly charged small molecules include inositol-1,3,4,5,6-pentakisphosphate (IP5) and IP6. IP6 binds to numerous host cell proteins and regulates diverse biological processes, including chromatin remodeling (11), mRNA nuclear export (12, 13), platelet aggregation (14), prion propagation (15), and circadian rhythm (16). IP6 also binds to the HIV-1 capsid *in vitro* and stabilizes the hexameric CA lattice. It associates with the center of the CA hexamer, forming ionic interactions with the six Arg18 side chains residing within the hexamer pore formed by the CA N-terminal domains (17, 18). In endogenous reverse transcription (ERT) reactions with purified HIV-1 cores, the addition of IP6 protected the newly synthesized viral DNA from degradation by exogenously added DNase I *in vitro*, suggesting that the viral capsid can provide a barrier to access of the viral genome (18). IP6 is incorporated into budding HIV-1 particles via an interaction with a distinct site in the assembling Gag lattice, near the CA-SP1 junction (17). It has been proposed that during maturation, IP6 is released upon proteolytic cleavage of Gag and subsequently associates with the mature capsid lattice and stabilizes it (17, 18). The critical importance of capsid stability in HIV-1 reverse transcription and infection, coupled with the relative biochemical instability of purified HIV-1 cores, makes this an appealing model. However, a role of IP6 in reverse transcription itself has not been established.

Although HIV-1 reverse transcription occurs efficiently in permissive target cells, *in vitro* assays of reverse transcription in permeabilized virions are typically inefficient for unclear reasons. In these ERT reactions, only a small fraction of viral genomes is converted into full-length double-stranded DNA molecules. Such reactions have frequently relied on the addition of detergents or other membrane-disrupting agents to suspensions of concentrated virions, thereby permitting access of deoxynucleoside triphosphates (dNTPs) to the viral core (19, 20). The addition of detergents may compromise ERT by destabilizing the viral capsid, resulting in dissociation of RT from the template and its diffusion out of the viral core (21). HIV-1 reverse transcription complexes (RTCs) isolated from acutely infected cells generally lack substantial quantities of the CA protein (22), suggesting that the capsid dissociates during cell permeabilization. The apparent fragility of HIV-1 cores and reverse transcription complexes has hampered biochemical studies of early events in HIV-1 infection, specifically reverse transcription and the role of the viral capsid in this process.

To address this problem, we identified detergent-free experimental conditions in which HIV-1 cores purified from infectious virions undergo efficient reverse transcription. We show here that addition of the capsid-stabilizing cell metabolite IP6 markedly enhances the efficiency of reverse transcription by promoting the synthesis of full-length minus-strand DNA. IP6 also stabilized the association of the CA and RT proteins with HIV-1 cores, suggesting that the effect was mediated by capsid stabilization. Our results are consistent with a model in which the viral capsid promotes retention of a sufficient concentration of RT in association with the viral genome to ensure completion of reverse transcription.

## RESULTS

### Establishment of the ERT reaction using purified HIV-1 cores.

**FIG 1:**
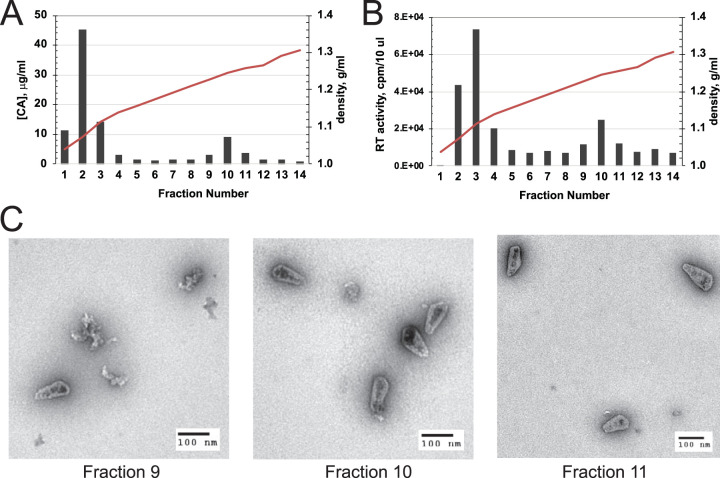
Characterization of purified HIV-1 cores. HIV-1 cores were isolated from concentrated virions by sucrose density gradient sedimentation. Gradient fractions were assayed for CA protein (A) and RT activity (B). (C) Electron micrographs from negatively stained samples of the three gradient fractions containing HIV-1 cores.

In an effort to improve the efficiency of the HIV-1 ERT reaction, we incubated samples of purified HIV-1 cores with dNTPs *in vitro* and analyzed the products by quantitative PCR (qPCR). For this purpose, we purified HIV-1 cores by a method involving ultracentrifugation of concentrated virions through a layer of Triton X-100 detergent into a sucrose density gradient. Under these conditions, the virions are exposed to the detergent for only a brief time, thereby preserving the integrity of the viral core. During centrifugation, HIV-1 cores sediment into the gradient, resulting in the removal of the detergent. Cores were detected in gradient fractions by p24 enzyme-linked immunosorbent assay (ELISA) for the CA protein (Fig. 1A), by assay for RT activity (Fig. 1B), and by negative-stain electron microscopy (Fig. 1C).

**FIG 2:**
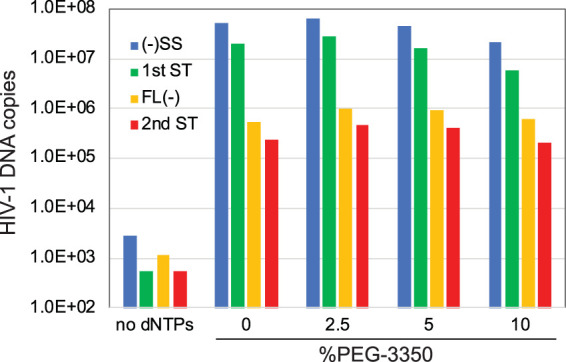
Representative results from an ERT experiment. HIV-1 cores were incubated for 16 h in a preliminary ERT reaction buffer containing the indicated concentrations of PEG3350. DNA was purified in the reactions and analyzed by qPCR for the indicated products of reverse transcription. This experiment was one of many early attempts to improve the efficiency of ERT.

In initial studies, we attempted to improve the reaction efficiency by varying the temperature, reaction time, and pH. We also tested polyethylene glycols, which have been previously reported to stimulate reverse transcription *in vitro* (23), and tested the effects of adding bovine serum albumin (BSA). After extended incubation at 37°C, the DNA products were purified and quantified for sequences corresponding to various stages of reverse transcription. An example of the results obtained in this type of experiment is shown in Fig. 2. qPCR analysis demonstrated that some of these parameters resulted in a modest increase in overall ERT efficiency (based on the ratio of 2nd-strand transfer products to minus-strand strong stop molecules). In particular, the addition of BSA appeared beneficial, while the inclusion of polyethylene glycol 3350 (PEG3350) resulted in no substantial improvement. Based on the quantitative analysis of each stage of reverse transcription, the overall efficiency of the reactions appeared to be limited by a marked attenuation in full-length minus-strand synthesis. In contrast, both strand transfer events were relatively efficient. These results indicated that the cores initiated reverse transcription but were unable to synthesize the complete minus strand. Of note, the reactions required prolonged incubation times, suggesting that the viral capsid dissociated during the incubation, resulting in a loss of RT, as previously reported (1).

### IP6 promotes efficient minus-strand synthesis in ERT reactions.

**FIG 3:**
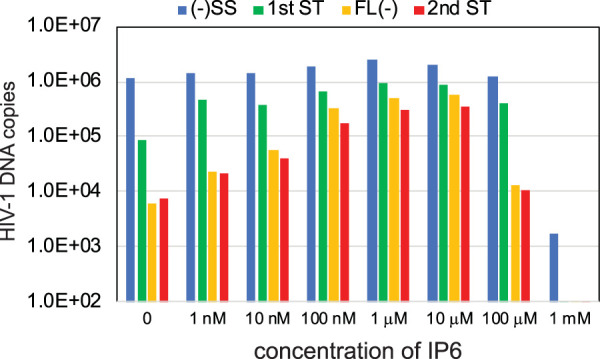
IP6 markedly stimulates ERT *in vitro* by enhancing minus-strand synthesis. ERT reaction mixtures containing the indicated concentrations of IP6 were incubated at 37°C for 16 h, and DNA products were purified and quantified.

The cell metabolite IP6 was recently reported to bind to the HIV-1 capsid *in vitro* and to promote the assembly of recombinant CA protein into capsid-like structures (17, 18). Adding IP6 to permeabilized HIV-1 particles resulted in protection of newly synthesized viral DNA from degradation by DNase I. By stabilizing the viral capsid, IP6 may prevent access of the nuclease to the nascent viral DNA. However, in that study, addition of IP6 did not enhance ERT reactions performed in the absence of DNase I. To test whether capsid stabilization by IP6 can enable efficient ERT, we performed reactions in the presence of a range of IP6 concentrations (Fig. 3). We observed a marked enhancement of full-length minus-strand synthesis and increased overall efficiency of the reaction in the presence of low-micromolar concentrations of IP6. These results indicate that IP6 increases the efficiency of ERT by promoting the synthesis of late-stage products.

**FIG 4:**
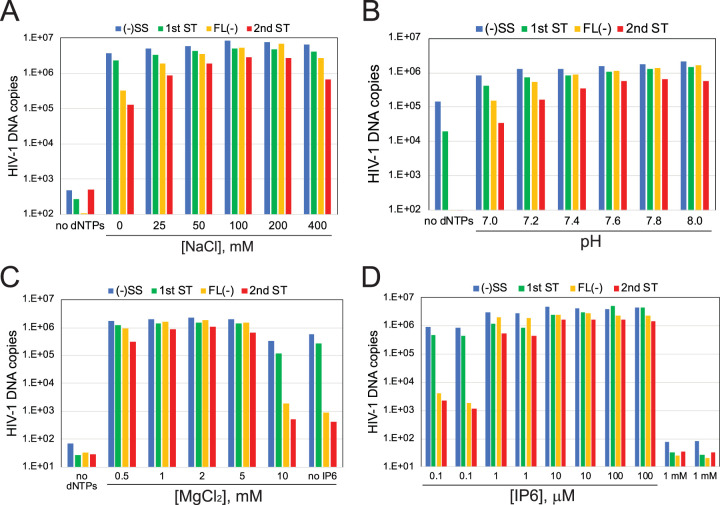
Optimization of the ERT reaction. (A to C) ERT reaction mixtures containing 10 μM IP6 under the indicated conditions were incubated and analyzed for HIV-1 DNA products. The variables were NaCl concentration (A), pH in reaction mixtures containing 150 mM NaCl (B), and MgCl_2_ concentration in reaction mixtures at pH 7.6 with 150 mM NaCl (C). (D) Titration of IP6 in ERT reaction mixtures under optimized conditions. Duplicate reaction mixtures were incubated for 16 h at 37°C in 50-μl reaction mixtures containing HIV-1 cores in a buffer containing 10 mM Tris-HCl, pH 7.6, 150 mM NaCl, 2 mM MgCl_2_, 0.5 mM DTT, 1 mg/ml BSA.

Following this observation, we optimized several parameters in reaction mixtures containing 10 μM IP6, including NaCl and MgCl_2_ concentrations and pH (Fig. 4A to C). Thus, we identified conditions for efficient ERT: 10 mM Tris-HCl, pH 7.6, 150 mM NaCl, 2 mM MgCl_2_, 1 mg/ml BSA, 0.5 mM dithiothreitol (DTT), and 10 μM IP6, with incubation at 37°C for 16 h. Under these conditions, we reproducibly observed conversion of 35 to 40% of the minus-strand strong stop products into molecules that also contained HIV-1 sequences that are synthesized only after the second-strand transfer step. This was the maximum ERT efficiency we observed in multiple experiments with different preparations of cores. Titration of IP6 under these conditions resulted in a broad optimum in the range of 10 to 100 μM, with strong inhibition of ERT occurring in reaction mixtures containing 1 mM IP6 (Fig. 4D).

### Kinetics of the ERT reaction.

**FIG 5:**
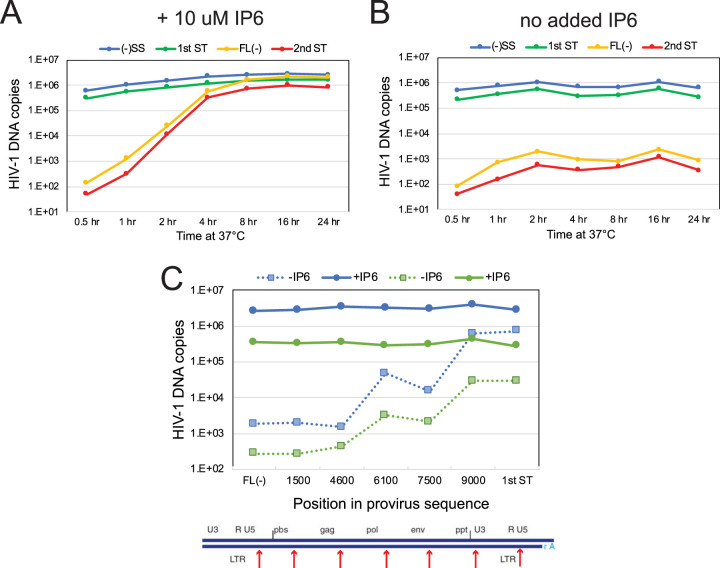
Time course of ERT in the presence and absence of IP6. ERT reaction mixtures containing or lacking 10 μM IP6 were incubated for the indicated times and subsequently analyzed for various HIV-1 DNA products by qPCR. (A) Reaction mixtures containing IP6; (B) reaction mixtures lacking IP6. Values represent averages from duplicate ERT reactions. (C) Quantification of products in 16-h reactions for sequences spanning the viral genome in reaction mixtures containing or lacking IP6. The blue and green symbols represent values from pairs of ERT reactions from two different experiments. Dashed lines connect values from ERT reaction mixtures lacking IP6. Results shown are from one of two independent experiments.

We also analyzed the rates of product formation in ERT reactions under the optimized conditions. Minus-strand strong stop and 1st-strand transfer products were synthesized rapidly in reaction mixtures containing IP6, reaching half-maximal values within 2 h (Fig. 5A). In contrast, synthesis of full-length minus-strand and 2nd-strand transfer products required 4 to 8 h to reach half-maximal values. In reaction mixtures lacking IP6, both early product species were also 50% complete after 2 h but declined slightly, suggesting the possibility of partial degradation (Fig. 5B). In reaction mixtures lacking IP6, low levels of late products were produced at 2 h and declined thereafter. Late products were then slightly increased at the 16-h time point, suggesting that degradation competes with ongoing synthesis. While IP6 stimulated the synthesis of early products up to 10-fold, the effect on late-stage reverse transcripts was profound, enhancing product accumulation by several thousandfold (compare Fig. 4A to B). PCR quantification of the 16-h ERT products generated in the absence of IP6 using primers spanning the genome revealed that the extension of the minus strand was impaired, with few DNA products longer than 3 kb accumulating in the reaction mixtures (Fig. 5C).

### HCB stimulates ERT.

**FIG 6:**
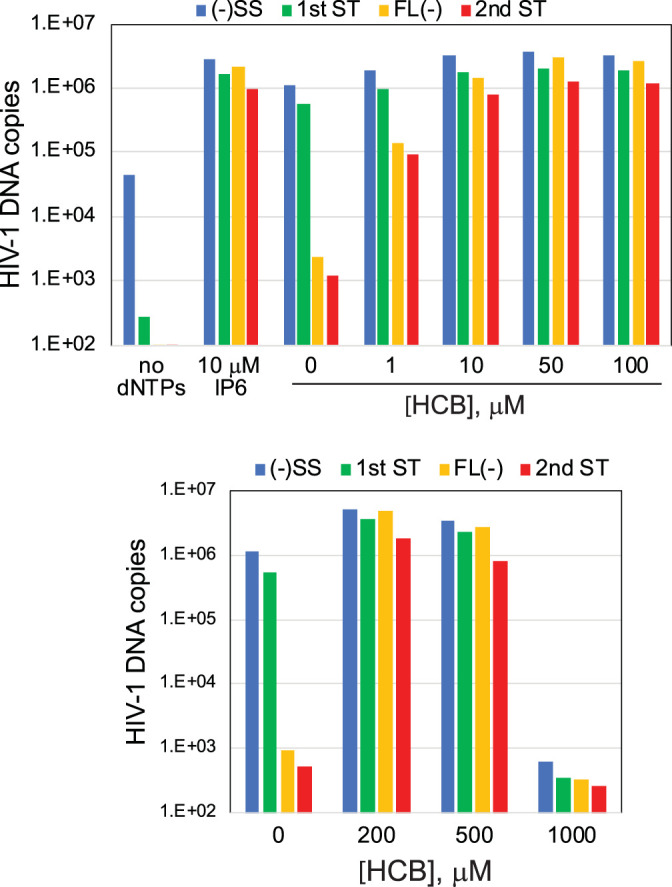
Capsid-stabilizing compound HCB also stimulates ERT. Reaction mixtures containing the indicated concentrations of HCB were incubated for 16 h at 37°C, and the products were analyzed by qPCR. Results shown are representative of two independent experiments.

We also tested the synthetic hexavalent compound hexacarboxybenzene (HCB) in ERT reactions, owing to a previous report that HCB stabilizes HIV-1 cores *in vitro* (24). The addition of HCB markedly enhanced minus-strand synthesis at an optimal concentration of ∼100 μM, resulting in efficient ERT (Fig. 6A). HCB inhibited all stages of ERT when present at a concentration of 1 mM (Fig. 6B), consistent with a previous study reporting inhibition of ERT by 20 mM HCB (24).

**FIG 7:**
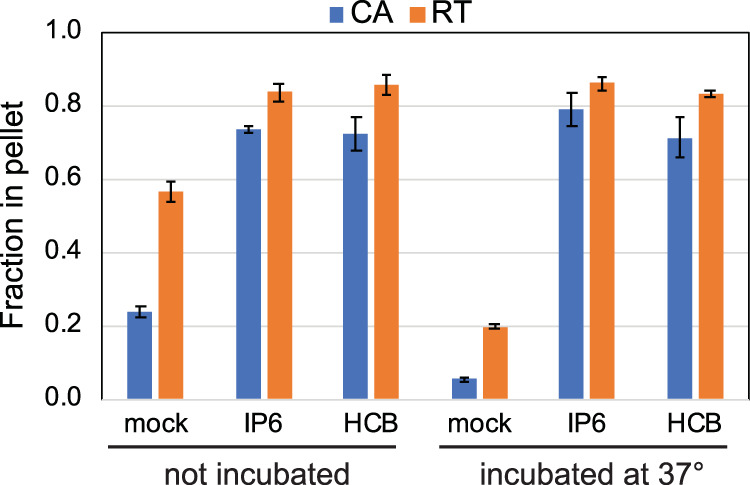
IP6 and HCB stabilize viral cores during reverse transcription. Shown are ERT reaction mixtures containing no additive, 10 μM IP6, or 100 μM HCB, not incubated or incubated at 37°C for 6 h. Reaction mixtures were diluted 10-fold with reaction buffer, the cores were pelleted by ultracentrifugation, and the pellets and supernatants were analyzed for CA and RT activity. Shown is the fraction of the total CA and RT activity in the pellets. The values shown are the average values of duplicate reactions from one of two independent experiments, which showed similar outcomes.

To determine whether IP6 and HCB stabilize the HIV-1 capsid during ERT, we analyzed the quantity of HIV-1 CA and RT released from viral cores in reaction mixtures containing these compounds. Following a 6-h incubation, the reaction mixtures were diluted with cold buffer and subjected to ultracentrifugation to separate the soluble proteins from those that remained core associated. Quantification of the fraction of the total CA present in the pellets revealed higher levels of pelletable CA protein in reaction mixtures containing IP6 or HCB, indicating that the viral capsid was stabilized by the compound (Fig. 7). Similarly, assays of RT activity in the supernatants and pellets showed that IP6 and HCB increased the levels of pelletable RT in the reaction mixtures. Collectively, these results suggest that the enhancing effects of IP6 and HCB on ERT result from stabilization of the viral capsid.

### Cores from an HIV-1 mutant with a hyperstable capsid undergo efficient ERT in the absence of added capsid stabilizers.

**FIG 8:**
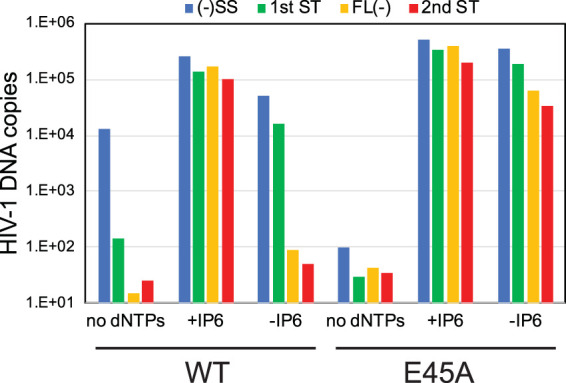
Cores from the E45A HIV-1 mutant, which contains a hyperstable capsid, are less dependent on IP6 or HCB for ERT. Cores were purified from HIV-1 particles that had been produced by transfection of 293T cells. ERT reactions were performed with and without added IP6 or HCB. Results shown are representative of two independent experiments.

We also asked whether genetic stabilization of the HIV-1 capsid affects the dependence of ERT on IP6. For this purpose, we isolated cores from HIV-1 particles containing the capsid-stabilizing CA substitution E45A. This mutant is competent for reverse transcription in target cells but exhibits impaired infectivity owing to inefficient nuclear entry and integration (4). Cores from the E45A mutant are hyperstable *in vitro*, as inferred from their increased levels of core-associated CA and slower dissociation of CA during incubation at 37°C (1). In reaction mixtures containing IP6, the mutant cores exhibited efficient ERT, as did those from the wild type (Fig. 8). In reaction mixtures lacking IP6, E45A cores produced approximately 20% of the late-stage products relative to parallel reaction mixtures containing IP6. This is in stark contrast to reactions with wild-type cores, in which synthesis of late products was less than 0.1% of that observed in reaction mixtures containing IP6. These results indicate that E45A mutant cores are capable of synthesizing substantial quantities of late reverse transcripts in the absence of capsid-stabilizing agents, further indicating that the ERT-stimulating activity of IP6 results from stabilization of the viral capsid.

### The capsid-targeting compound PF74 inhibits ERT in a concentration-dependent manner.

**FIG 9:**
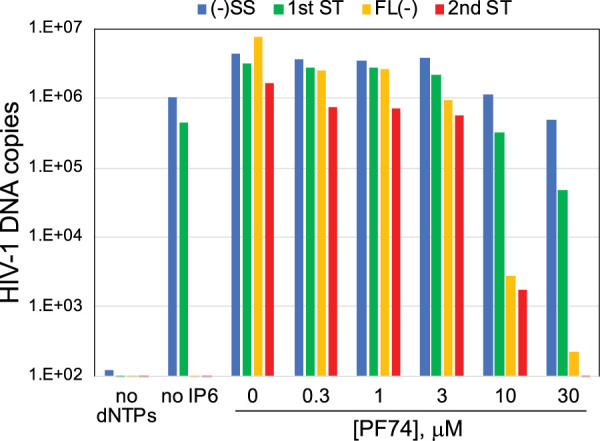
Capsid-targeting antiviral compound PF74 inhibits ERT. Optimized ERT reaction mixtures containing the indicated concentrations of PF74 were incubated for 16 h. DNA products were purified and analyzed for HIV-1 sequences by qPCR. These results are representative of 3 independent experiments.

Finally, we tested the effects of the capsid-targeting antiviral compound PF74. PF74 binds to a site in the CA hexamer that is distinct from that bound by IP6 (25, 26). When present during HIV-1 infection at concentrations of 10 μM and above, PF74 inhibits reverse transcription and destabilizes the viral capsid (27). Addition of 10 μM PF74 inhibited ERT in reaction mixtures containing IP6 (Fig. 9), further linking capsid function to ERT efficiency. In contrast, addition of 1 μM PF74 did not substantially inhibit ERT, consistent with previous reports that at low concentrations PF74 inhibits HIV-1 infection mainly by affecting nuclear entry and integration (28–30). These results further underscore the critical importance of HIV-1 capsid stability in ERT.

## DISCUSSION

In this study, we observed that the addition of IP6 stabilizes HIV-1 cores and markedly enhances the efficiency of reverse transcription *in vitro*. Mallery and coworkers had previously shown that IP6 stabilizes purified HIV-1 cores and protects the products of ERT from degradation by added DNase I, suggesting that the capsid provides a barrier to access to the synthesized DNA (18). In that study, IP6 did not appear to substantially alter the quantity of DNA products in the absence of added DNase. We observed the inhibition of ERT at a high concentration of IP6 (1 mM). Although the mechanistic basis for inhibition is unknown, we suspect it results from acidification of the reactions by the nonneutralized IP6 solution. In contrast, the addition of lower concentrations of IP6 resulted in a nearly quantitative conversion of the initial minus-strand products to full-length molecules. The observed enhancement resulted from a roughly thousandfold increase in minus-strand synthesis together with modest enhancements at both strand transfer steps. We conclude that IP6 dramatically enhances the completion of minus-strand synthesis. The maximum efficiency of these reactions was ∼40% based on the ratio of 2nd-strand transfer products relative to minus-strand strong stop molecules. It is intriguing that the major effect of IP6 was observed at minus-strand synthesis rather than the strand transfers, which have been reported to occur slowly in model systems. In contrast, both strand transfer events occurred rapidly and efficiently in the ERT reactions (Fig. 5). These results indicate that purified cores can, under appropriate conditions, undergo efficient reverse transcription *in vitro* in the absence of added host proteins, as was described in early studies of murine and avian retroviruses (31–33).

IP6 and the synthetic compound HCB also stabilized the association of CA and RT with viral cores, as previously observed in imaging studies of permeabilized virions. Thus, a plausible mechanistic conclusion is that the observed enhancement of ERT results from capsid stabilization, because most HIV-1 mutants containing intrinsically unstable capsids are impaired for reverse transcription in target cells (1). We also observed that mutant HIV-1 cores with hyperstable capsids synthesized substantial quantities of full-length minus-strand DNA in reaction mixtures lacking IP6. Finally, we observed that the capsid-destabilizing HIV-1 inhibitor PF74 inhibited ERT even in the presence of IP6. Together, these observations support a capsid stabilization mechanism for enhancement of ERT by IP6. In addition, studies involving computational modeling have suggested that IP6 binding to the HIV-1 capsid also facilitates the import of dNTPs into the viral core, an effect that may contribute to the enhancement of ERT observed in our experiments (34, 35).

How might stabilization of the viral capsid help promote the completion of reverse transcription? We observed that IP6 caused a profound enhancement of minus-strand DNA synthesis, with lesser effects on the other steps that were quantified, including both strand transfers. In earlier work, our group showed that spontaneous uncoating of purified HIV-1 cores *in vitro* is characterized by dissociation of both CA and RT from the viral core (1). We suggest that the capsid acts as a container to retain RT during synthesis of the long (∼9-kb) minus-strand DNA. HIV-1 particles are estimated to contain approximately 50 molecules of RT (7), with about 20% of the active enzyme copurifying with cores (Fig. 1B). While completion of reverse transcription is theoretically possible with a single molecule of the enzyme, the relatively low processivity of the enzyme suggests that RT must repeatedly rebind the template to synthesize full-length viral DNA. By preserving the association of RT with the core, the viral capsid may ensure that RT is maintained at a sufficient concentration to allow completion of the reaction. Nonetheless, this “container model” does not exclude additional possible functions of the capsid in reverse transcription, including providing a molecular scaffold on which the reaction may occur.

IP6 is a natural metabolite that is present in mammalian cells at concentrations ranging from 40 to 90 μM (36), coinciding well with the ERT-enhancing effects we observed in the present study. Therefore, one could expect that HIV-1 infection would be strongly dependent on target cell levels of IP6 and/or the related metabolite IP5. However, a recent study reported that ablation of cell expression of host proteins that synthesize these compounds does not appreciably influence their susceptibility to HIV-1 infection (37). These studies were performed in a transformed epithelial cell line, and it will be important to determine whether these inositol phosphates influence HIV-1 infection of physiologically relevant target cells. Additionally, purified HIV-1 cores appear to contain substantial quantities of bound IP6 (18), suggesting that the viral capsid is stabilized by the IP6 that is captured during virus particle assembly.

Reverse transcription is thought to promote HIV-1 uncoating in target cells, and it leads to physical changes in HIV-1 cores *in vitro* (38–40). The ERT reaction described here mimics the kinetics of reverse transcription observed during synchronous HIV-1 infection of target cells (41), albeit without the initial lag phase, which presumably results from the requirement for virus fusion. Moreover, the reaction appears to be highly synchronous, with early DNA products appearing rapidly and the subsequent products accumulating only after a substantial delay. The experimental system described here should enable structural and biochemical studies of native HIV-1 reverse transcription complexes and analysis of the role of the viral capsid in the process, including high-resolution analysis of the effects of reverse transcription on the structure of the viral core. The assay should also help further define the mechanism of action of capsid-targeting inhibitors, such as PF74. Finally, efficient ERT reactions may also permit the generation of substantial quantities of pure and active HIV-1 PICs *in vitro* for biochemical studies of HIV-1 integration.

## MATERIALS AND METHODS

### Chemicals, cells, and plasmids.

IP6 was purchased as a 1.1 M solution from TCI America (catalog no. P0409). Tris, mellitic acid (HCB), and PEG3350 were purchased from Sigma. Deoxynucleoside triphosphates (dNTPs) were purchased from New England Biolabs as 100 mM solutions. BSA was purchased from RPI (catalog no. A30075). The antiviral compound PF74 was synthesized and purified by the Chemical Synthesis Core of the Vanderbilt University Institute for Chemical Biology. For qPCR, the Maxima SYBR green/ROX qPCR master mix (2×), from ThermoScientific (catalog no. K0233), was used. Custom oligodeoxyribonucleotides were purchased from Integrated DNA Technologies.

### Purification and analysis of HIV-1 cores.

For most experiments, HIV-1 cores were purified from 200 ml of virus particles collected from infected MT-4 cells. Cultured MT-4 cells (1 × 10^7^) were pelleted and resuspended in 50 ml of medium. Cultures were inoculated wild-type HIV-1 particles produced by transfection of 293T cells with the wild-type R9 proviral construct. A quantity of the virus stock, corresponding to approximately 5 μg of p24, was added to the MT-4 cultures with DEAE-dextran at a final concentration of 20 μg/ml. The following day, the cells were pelleted and resuspended in 200 ml of fresh culture medium. The cultures were examined daily for signs of virus-induced cytopathicity, and at day 4 to 5 after inoculation, the cultures were centrifuged to remove cells and cell debris. The virus-containing culture supernatants were clarified by filtration and concentrated by ultracentrifugation at 32,000 rpm in a Beckman SW32.1Ti rotor at 4°C for 3 h. Concentrated virions were resuspended in a total volume of 0.5 ml.

For the experiments shown in Fig. 8, HIV-1 cores were purified from virions produced by transfection of 293T cells, as previously described (42), with the following modifications. Four million 293T cells were transfected with 10 μg of R9 and R9.E45A plasmid DNAs using polyethyleneimine (43). The next day, cultures were washed and replenished with fresh medium. The following day, the culture supernatants were collected, clarified by filtration through a 0.45-μm vacuum filtration device, treated with 20 μg/ml DNase I and 10 mM MgCl_2_ at 37°C for 1 h to eliminate residual carryover plasmid DNA, and then concentrated for purification of viral cores.

To purify cores, concentrated HIV-1 particles were subjected to centrifugation through a layer of Triton X-100 (1%, vol/vol) into a linear sucrose density gradient, as previously described (42). Fractions (1 ml) were collected and assayed for CA protein by p24 ELISA and RT activity. The dense fractions corresponding to HIV-1 cores typically contained approximately 15% of the total CA protein in the gradients. Fractions containing HIV-1 cores were aliquoted, flash-frozen in liquid nitrogen, and stored at −80°C for ERT reactions and other assays.

Negative-stain electron microscopy of purified cores was performed by applying 3- to 5-μl samples of core preparations directly to glow-discharged carbon-coated copper electron microscopy grids. Following 1 min of adherence, the excess liquid was removed by wicking, and the grids were inverted for 1 min onto two consecutive droplets containing uranyl formate stain, followed by two consecutive inversions onto water droplets. The liquid was carefully removed by wicking, and the grids were air-dried and imaged in a Morgagni electron microscope. Images were captured using a charge-coupled device camera.

### Assays of ERT using purified HIV-1 cores.

ERT reactions were performed in 50-μl volumes containing 10 mM Tris-HCl, pH 7.6, 150 mM NaCl, 2 mM MgCl_2_, 0.5 mM DTT, 0.1 mM (each) 4 dNTPs, 1 mg/ml BSA, and various concentrations of additives, including IP6, HCB, PEG3350, and PF74. Reactions were normally incubated at 37°C for 16 h, after which the DNA was extracted using a silica gel-based method (44) and eluted in water. The products were quantified by qPCR with SYBR green detection using the primers listed in Table 1.

**TABLE 1:** Primers used for quantification of HIV-1 reverse transcripts

Stage of reverse transcription^*a*^	Primer sequence	Position in provirus sequence (orientation^*b*^)
Minus-strand strong stop	5′-GCCTCAATAAAGCTTGCCTTGA-3′	522–543 (F)
	5′-TGACTAAAAGGGTCTGAGGGATCT-3′	592–615 (R)
First-strand transfer	5′-GAGCCCTCAGATCCTGCATAT-3′	9493–9513 (F)
	5′-CCACACTGACTAAAAGGGTCTGAG-3′	9682–9705 (R)
Full-length minus strand	5′-CTAGAACGATTCGCAGTTAATCCT-3′	909–932 (F)
	5′-CTATCCTTTGATGCACACAATAGAG-3′	1041–1065 (R)
Second-strand transfer	5′-TGTGTGCCCGTCTGTTGTGT-3′	557–576 (F)
	5′-GAGTCCTGCGTCGAGAGAGC-3′	677–696 (R)
Minus strand (8900)	5′-AGGGAAAGAATGAGACGAGC-3′	8835–8854 (F)
	5′-GCTACTTGTGATTGCTCCATG-3′	8904–8924 (R)
Minus strand (7500)	5′-TGGAGTACTGAAGGGTCAAATAAC-3′	7412–7435 (F)
	5′-ACTTCCTGCCACATGTTTATAAATTG-3′	7478–7503 (R)
Minus strand (6000)	5′-TTGTTTCATGACAAAAGCCTTAGG-3′	5937–5960 (F)
	5′-GTCTGACTGTTCTGATGAGCTC-3′	5999–6020 (R)
Minus strand (4500)	5′-GGCAGCTAGATTGTACACATTTAG-3′	4441–4434 (F)
	5′-TGCTGGAATTACTTCTGCTTCT-3′	4481–4502 (R)
Minus strand (2900)	5′-GCAGGGTTAAAACAGAAAAAATCAG-3′	2841–2865 (F)
	5′-CCTGAAGTCTTTATCTAAGGGAACTG-3′	2899–2924 (R)
Minus strand (1400)	5′-ACCATGCTAAACACAGTGGG-3′	1345–1364 (F)
	5′-AGCTTCCTCATTGATGGTCTC-3′	1396–1416 (R)

a Numbers in parentheses represent the approximate positions of the corresponding qPCR amplicons in the HIV-1 provirus sequence.

b F, forward; R, reverse.

Quantitative PCRs were performed in 20-μl volumes with a Stratagene Mx3000p real-time thermal cycler according to the following program: 95°C for 10 min (1 cycle), followed by 95°C for 30 s, 55° for 1 min, and 72° for 1 min (40 cycles). DNA copy numbers were interpolated from standard curves of threshold cycle (*C_T_*) values generated from reaction mixtures containing dilutions of R9 proviral plasmid DNA, performed in parallel.

### Uncoating assays.

ERT reaction mixtures (50 μl) were incubated at 37°C for 6 h, diluted with 450 μl cold reaction buffer lacking dNTPs, and subjected to centrifugation for 30 min at 100,000 × *g* in a Beckman TLA-55 rotor. Control reactions were diluted and pelleted immediately after mixing. The supernatants were withdrawn and transferred to clean tubes, and the pellets were resuspended in ERT reaction buffer (0.5 ml). The supernatants and pellets were assayed for CA protein by p24 ELISA (45) and for RT activity using an exogenous primer-template assay, as previously described (46). The resulting values were used to calculate the fraction of pelleted CA and RT activity in the samples.

## ADDENDUM IN PROOF

During the review of the manuscript, enhancement of HIV-1 ERT by IP6 in permeabilized virions was reported in reference 47.
